# Genomic alterations of oligodendrogliomas at distant recurrence

**DOI:** 10.1002/cam4.6327

**Published:** 2023-08-02

**Authors:** Guanzheng Liu, Chaojie Bu, Guangzhong Guo, Zhiyue Zhang, Zhiyuan Sheng, Kaiyuan Deng, Shuang Wu, Sensen Xu, Yage Bu, Yushuai Gao, Meiyun Wang, Gang Liu, Lingfei Kong, Tianxiao Li, Ming Li, Xingyao Bu

**Affiliations:** ^1^ Department of Neurosurgery Zhengzhou University People's Hospital, Henan Provincial People's Hospital Zhengzhou China; ^2^ Juha International Central Laboratory of Neurosurgery Henan Provincial People's Hospital Zhengzhou China; ^3^ Department of Radiology Henan Provincial People's Hospital Zhengzhou China; ^4^ Department of Center for Clinical Single Cell Biomedicine, Department of Oncology, Clinical Research Center, Henan Provincial People's Hospital Zhengzhou University People's Hospital Zhengzhou China; ^5^ Department of Pathology Henan Provincial People's Hospital Zhengzhou China

**Keywords:** ctDNA, gene evolution, oligodendroglioma, PI3K/AKT signaling pathway, SHh signaling pathway

## Abstract

**Background:**

Oligodendroglioma is known for its relatively better prognosis and responsiveness to radiotherapy and chemotherapy. However, little is known about the evolution of genetic changes as oligodendroglioma progresses.

**Methods:**

In this study, we evaluated gene evolution invivo during tumor progression based on deep whole‐genome sequencing data (ctDNA). We analyzed longitudinal genomic data from six patients during tumor evolution, of which five patients developed distant recurrence.

**Results:**

Whole‐exome sequencing demonstrated that the rate of shared mutations between the primary and recurrent samples was relatively low. In two cases, even well‐known major driver mutations in CIC and FUBP1 that were detected in primary tumors were not detected in the relapse samples. Among these cases, two patients had a conversion from the IDH mutation in the originating state to the IDH1 wild state during the process of gene evolution under chemotherapy treatment, indicating that the cell phenotype and genetic characteristics of oligodendroglioma may change during tumor evolution. Two patients received long‐term temozolomide (TMZ) treatment before the operation, and we found that recurrence tumors harbored mutations in the PI3K/AKT and Sonic hedgehog (SHh) signaling pathways. Hypermutation occurred with mutations in MMR genes in one patient, contributing to the rapid progression of the tumor.

**Conclusion:**

Oligodendroglioma displayed great spatial and temporal heterogeneity during tumor evolution. The PI3K/AKT and SHh signaling pathways may play an important role in promoting treatment resistance and distant relapse during oligodendroglioma evolution. In addition, there was a tendency to increase the degree of tumor malignancy during evolution. Distant recurrence may be a later event duringoligodendroglioma progression. ClinicalTrials.gov, Identifier: NCT05512325.

## 1 INTRODUCTION

The recently updated World Health Organization (WHO) classification of central nervous system (CNS) neoplasms incorporated more detailed molecular information into the definition of some CNS tumors in 2021. Among these CNS tumors, oligodendroglioma was molecularly defined as a glioma with IDH‐mutant and 1p/19q co‐deletion.^1^ Oligodendrogliomas with 1p/19q co‐deletion tend to retain their cell phenotypes and genetic alterations,^2^ demonstrate a marked chemotherapy response and long‐term survival.^3^, ^4^ However, recurrence of oligodendroglioma is virtually inevitable under the chemotherapy treatment, and the identification of gene evolution in oligodendroglioma has important clinical implications.

Recently, it has been determined that recurrent genetic alternations occur simultaneously with the deletion of 1p/19q co‐deletion. Some tumors with 1p/19q co‐deletion are accompanied by mutations in CIC and FUBP1, but these mutations do not seem essential for the establishment of the histological and clinical features of oligodendroglioma.^5^ Oligodendroglioma has longer progression‐free survival and a lower tendency to progress into a more aggressive tumor phenotype.^6^ However, the molecular mechanisms that underlie such behaviors are not well known. Oligodendroglioma also displays remarkable spatial and temporal heterogeneity that is known to be related to tumor evolution.^7^ MGMT promoter methylation is a late event during oligodendroglioma evolution, and their chemosensitivity is not necessarily related to MGMT methylation status.^2^ The methylation landscape changes dynamically during the course of malignant progression.^8^ A previous study demonstrated that their malignancies were rarely promoted by additionally acquired mutations or genomic aberrations during tumor evolution.^9^ Oligodendrogliomas have a tendency to retain their cell phenotypes and genetic profiles.^2^ Such molecular evolution features may account for the clinically benign characteristics of oligodendroglioma. However, in our study, oligodendrogliomas were invariably found to progress, and distant recurrences occurred more frequently under long‐term TMZ treatment. Oligodendrogliomas with metastases in bone and scalp have been previously described.^10^, ^11^ To gain insight into the molecular mechanism underlying this behavior of oligodendroglioma, we explored the dynamic gene evolution in vivo during oligodendroglioma progression under TMZ adjuvant chemotherapy.

In our preliminary study, we reported that tumor in situ fluid (TISF) is a novel clinical source for real‐time genomic profiling of glioma.^12^ The shared mutation analysis has shown that TISF‐DNA data were of high quality, based on the high concordance between TISF and tissue samples. TISF‐DNA can depict the genetic signatures during glioma evolution after surgery, which may be more sensitive than CSF‐ctDNA.^13^ Our preliminary work has verified TISF‐DNA validity, which can delineate the recurrent genetic signatures of gliomas, and lay a foundation for us to understand the gene evolution of oligodendroglioma under TMZ treatment.

## 2 MATERIALS AND METHODS

### 2.1 Patient characteristics

We prospectively collected tumor specimens, matched TISF samples, and blood samples from 11 patients with oligodendroglioma, World Health Organization (WHO) grade II‐III, with IDH mutation and 1p19q co‐deletion. All patients were treated at Henan Provincial People's Hospital in China from July 2014 to January 2022. This work was supported by the Zhengzhou University, and all patients provided written informed consent to participate in the study and for use of their clinical data and specimens for research. This study was performed according to the research proposals approved by the Institutional Review Board of Henan Provincial People's Hospital and the Ethics Committee of the Zhengzhou University (approval number 2122432055). We reclassified all tumors according to the 2021 update to the WHO classification of tumors of the central nervous system.^2^ Histopathologic classification and molecular information were reviewed and confirmed to correspond to oligodendroglioma. Relevant clinical information, including age, gender, tumor location, postoperative therapy, and others, was obtained. Table S1 shows a summary of the respective patient dates.

### 2.2 Sample collection

TISF samples were harvested as previously described.^12^ We obtained a small amount of TISF from the implanted reservoir (intraoperative retention). TISF is fluid in the local surgical cavity, which may support in situ detection of gene evolution during oligodendroglioma progression. At the same time, blood samples (5 mL) for germline DNA control were also acquired. ctDNA profiling from tumor tissues and TISF samples has utility for real‐time assessment of dynamic tumor evolution.

### 2.3 DNA extraction

Cell‐free DNA (cfDNA) was extracted from TISF samples using the Cell‐Free DNA Isolation Kit (Thermo Fisher Scientific). While glioma genomic DNA was extracted from fresh tumor tissue using the QIAamp DNA Tissue & Blood Kit (Qiagen). Control genomic DNA was prepared from paired blood samples using the QIAamp DNA Mini Kit (Qiagen). All DNA extractions were performed with the respective kit according to the manufacturer's protocol. The DNA concentration of each sample was measured using a Qubit assay kit (Thermo Fisher Scientific).

### 2.4 ctDNA sequencing

DNA sequencing was performed and analyzed by Beijing Genetron Health Technology Co., Ltd. Briefly, genomic DNA was PCR amplified using a 10 ng of genomic DNA primer pool as a template. Oligodendroglioma samples underwent high‐throughput exome sequencing with a targeted deep sequencing assay of 68 genes recurrently mutated in brain tumors (Genetron Health). Sequencing data were processed using the Torrent SuiteTM software 4.4 and quantified using the Torrent Mapping and Alignment Program (TMAP) algorithm (Life Technologies).The variants were detected using the Variant Caller Plugin v4.4 (Life Technologies). We considered a mutant allele fraction of ≥0.3% and a total of ≥4 reads detectable in samples. Finally, the alignment files were normalized and checked using deeptools and visualized with the Integrative Genomics Viewer (IGV) software. Resulting sequence reads were aligned against the reference DNA sequence, and variants were analyzed by using the Next GENe® v.2.3.4 software (SoftGenetics). All detected mutations were annotated for genes using ANNOVAR, Oncotator, and Vep.

### 2.5 1p/19q status

Determination of 1p/19q co‐deletion status was performed locally by dual‐color fluorescence in situ hybridization (FISH) analysis.^14^ Briefly, paraffin‐embedded tumor samples were sectioned and deparaffinized. The labeled probes mixed with Cot‐1 DNA (Life Technologies) were denatured and hybridized to sections. After washing with PBS, the sections were incubated in anti‐digoxigenin antibody and fluorescein isothiocyanate‐conjugated secondary antibody. Sections were mounted with mounting medium and viewed under a Zeiss microscope (CarlZeiss Microscopy LLC).

### 2.6 Statistical analysis

We compared the VAFs of mutations between the primary cancer and disease progression. Statistical analysis was performed using GraphPad Prism 8.0. Comparison between two samples was performed using an independent sample *t*‐test. *p* < 0.05 was considered statistically significant.

## 3 RESULTS

### 3.1 Characteristics of oligodendroglioma cases

**FIGURE 1:**
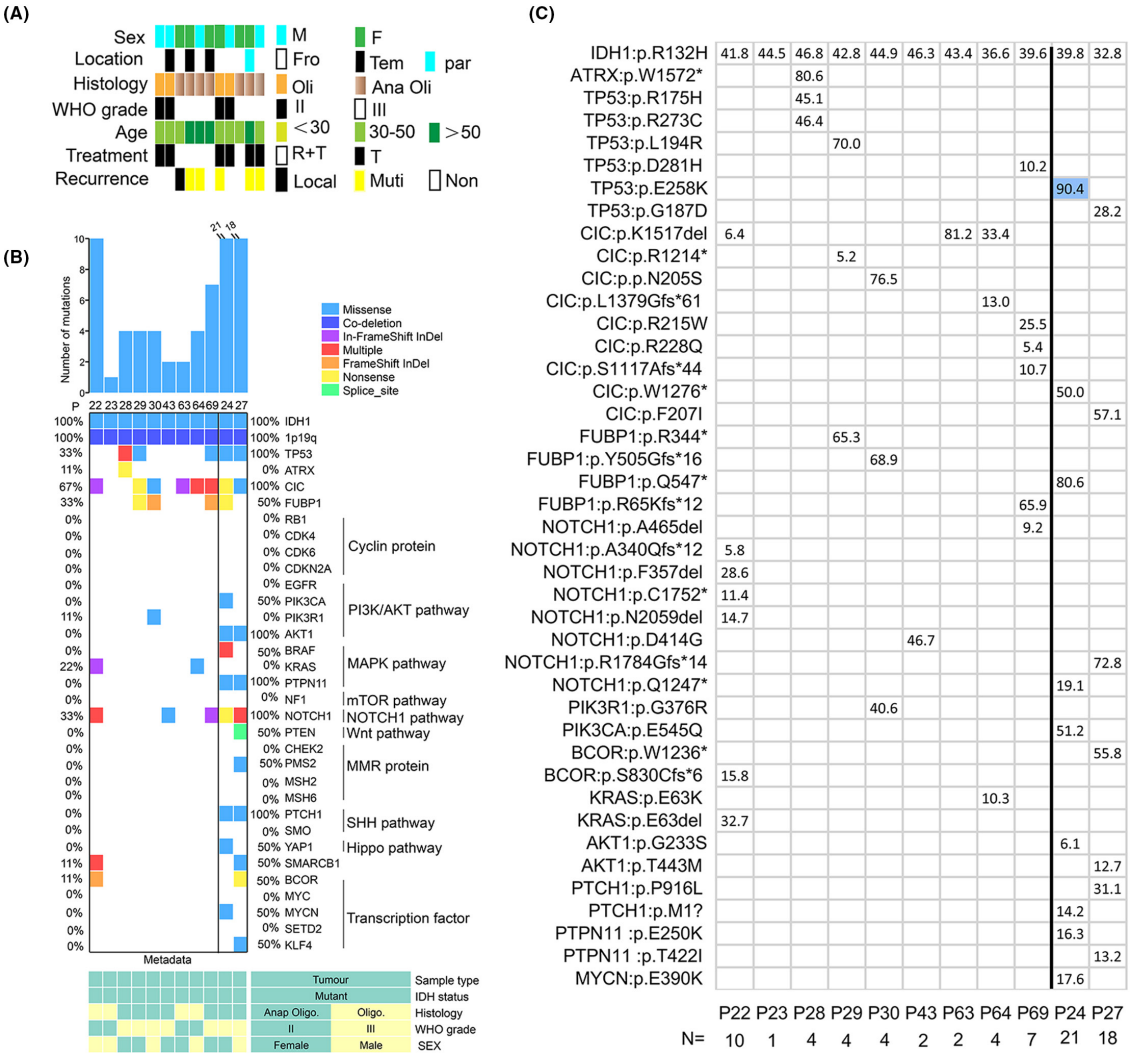
Characteristics of oligodendroglioma cases. (A) Overview of clinical parameters. (B) The spectrum of driver mutations in all patients was shown. Patient 24 and Patient 27 received long‐term TMZ treatment before the operation. (C) Distribution of mutational signatures associated with driver mutations.

To elucidate the mechanisms driving the evolution of oligodendroglioma under TMZ therapy, we analyzed exome sequencing data from 11 oligodendrogliomas. These cases harbored IDH1 mutations and 1p/19q co‐deletion: three patients with WHO grade II and eight patients with Grade III histology. After surgery, all patients were treated with TMZ treatment and five patients received radiotherapy treatments. Of the six patients who had a recurrence, one patient had local recurrence only and five patients had distant recurrence. Patients 24 and 27 received long‐term TMZ treatment before the surgery (Figure 1A).

We performed exome sequencing of 11 tumor samples and 11 matched normal blood samples. The sequencing data that include oligodendroglioma driver genes are summarized in Figure 1B. Among them, nine cases had no treatment before operation. Overall, our mutational analysis reveals both known and potentially driver gene mutations in oligodendroglioma. We observed major driver gene mutations known in oligodendroglioma, including CIC (6/9), FUBP1 (3/9), TP53 (3/9), Notch1 (3/9), KRAS (2/9), PIK3R1 (1/9), BOCR (1/9), SMARCB1 (1/9), and ATRX (1/9), accompanying with IDH‐mutant and 1p/19q co‐deletion (Figure 1B). We have no information for TERT promoter mutations due to exome sequencing. But long‐term exposure to therapeutic concentrations of TMZ before surgery, more mutations in SHh (Sonic hedgehog) and PI3K/AKT signaling pathways appeared, including PIK3CA (1/2), AKT1 (2/2), PTCH1 (2/2), and PTPN11 mutations (2/2). Even Patient 24 had a YAP1 mutation associated with the Hippo signaling pathway (Figure 1B). We show that a complex branching evolutionary pattern occurs in oligodendroglioma, and that clonal evolution can be influenced by TMZ treatment.

In addition, we further investigated mutation sites in primary tissues, which makes observation of timely tumor progression possible. R132H mutation of IDH1 is the most frequent genetic alteration in oligodendroglioma. IDH1:p.R132H mutation results in a gain of enzyme function and is thought to promote gliomagenesis.^15^ CIC:p.R215W mutation is a common mutation in oligodendroglioma, which leads to the loss of CIC protein function,^16^ while we observed CIC:p.K1517del was the most frequent mutation in our group (Figure 1C). In addition, under long‐term TMZ treatment, Patients 24 and 27 acquired different mutation sites in the same genes, such as TP53, NOTCH1, CIC, and FUBP1, in which mutations were observed in primary tumors. It is usually observed that the two patients acquired more mutations, and emerging evidence suggested that alterations in genes vary substantially, indicating spatiotemporal heterogeneity.

### 3.2 Recurrence mutation analysis

**FIGURE 2:**
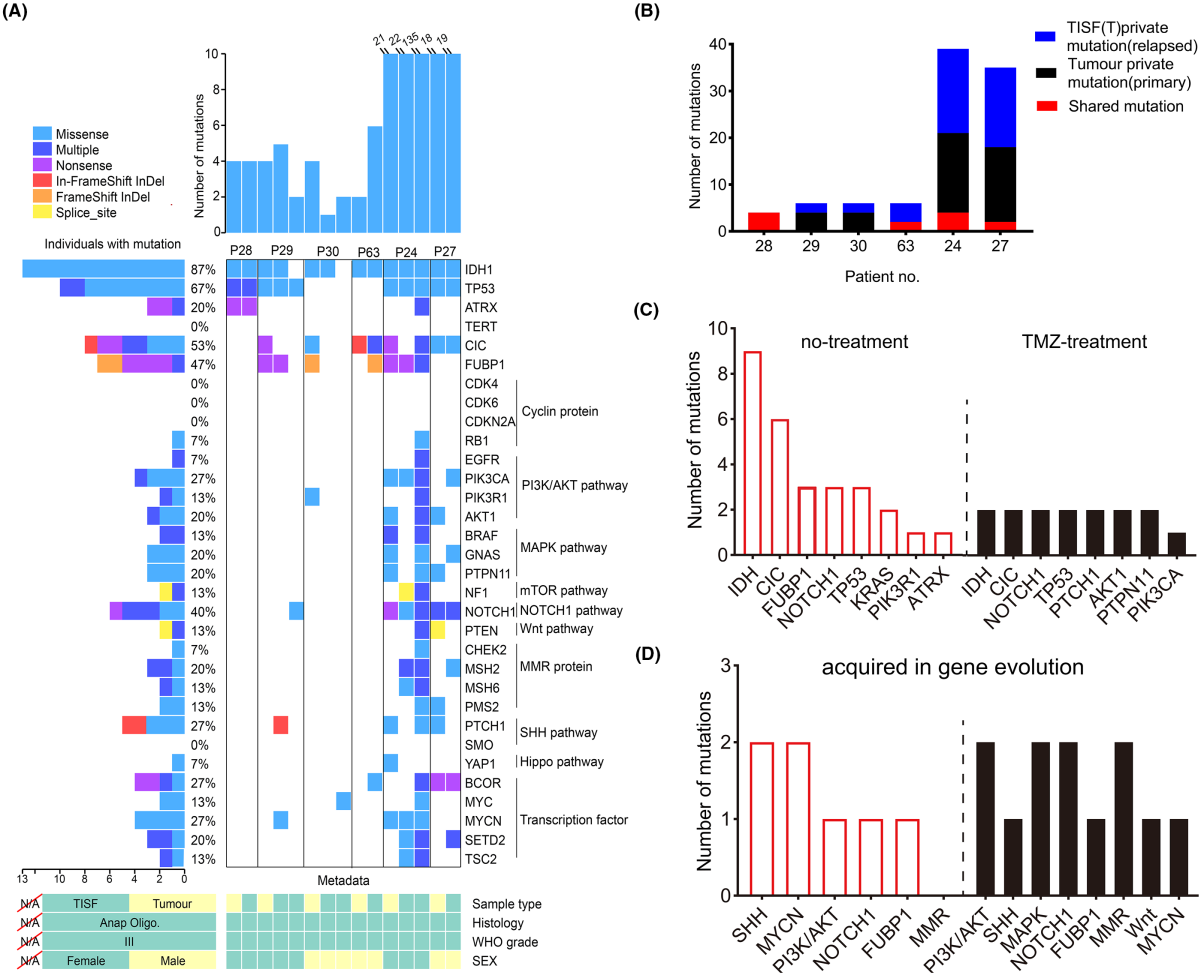
Recurrence mutation analysis. (A) Summary of the genomic profiles from the primary, local relapse, and distant relapse samples from two patients (Patient 29 and Patient 30). The number of non‐synonymous mutations, mutation profiles, and copy number alterations were shown from top to bottom of the panel. Genome profiling of the primary and relapse sample from Patient 28 and Patient 63 were depicted. Patient 24 and Patient 27 received long‐term TMZ therapy before the operation, gene landscapes were shown during progression. (B) All shared mutations and some private mutations number between primary tumor and relapse were shown. (C) The number of mutations related to driver genes were depicted. Patient 24 and Patient 27 received long‐term TMZ chemotherapy, and acquired additional mutations accompanied by driver genes. (D) The number of acquired mutations associated with signaling pathway were depicted.

We performed exome sequencing on tumor samples and matched real‐time TISF samples in vivo from six patients with recurrence. A total of 22 tumor and TISF samples were detected from these patients. The mutation list and detailed information can be found in the attached file Figure S1. Four patients had not received any treatment prior to surgery, and the average number of non‐synonymous mutations was 3.5 (ranging from 2 to 4). Two cases had received long‐term TMZ chemotherapy. The average number of non‐synonymous mutations in tumors was 19.5 (18, 21) (Figure 2A). And the retention rate, which is the ratio of primary tumor mutations retained in relapse tumors,^17^ was generally low (Figure 2B), with an average of 26.8% (ranging from 0 to 100), indicating that spatiotemporal heterogeneity may contribute to disease progression. Meanwhile, the gene evolution presented a large difference among different patients. Interestingly, there was one case in which the recurrent TISF sample shared the same mutations with the primary tumor (Figure 2A, Patient 28). Different mutations of the same gene, including CIC mutation in patient 63 and Notch1 mutation in Patient 24, were observed in recurrent TISF, which proved the convergent evolutionary model (Figure 2A). In order to infer the advantage of each mutation for tumor growth, we focused on the retained or acquired mutations in recurrent samples. In some cases, even the well‐known putative major driver gene mutations in CIC and FUBP1 detected in primary tumors were not detected in recurrence TISF samples (Patients 29 and 30), and the FUBP1 mutation was obtained with a low VAF of 0.3% in one case (Patient 63). It can be seen that CIC and FUBP1 mutations may have limited effects on promoting the progression and recurrence of oligodendroglioma. Furthermore, the IDH mutation status also changed, as it was not detected in recurrence samples (Patients 29 and 30) (Figure 2A). IDHmt‐to‐IDHwt transformation indicated that the cell phenotype and genetic characteristics of oligodendroglioma changed during tumor evolution, which promoted tumor progression into its more aggressive form.

Two patients received long‐term TMZ chemotherapy before surgery, and mutations in the SHh (PTCH1) and PI3K/AKT signaling pathways (AKT1, PIK3CA, PTPN11) were detected in two tumor tissues (Patients 24 and 27) (Figure 2C,D). Mutations in MMR genes were obtained (Patient 24 had MSH2 and MSH6 mutations, and Patient 27 had an MSH6 mutation), and hypermutation occurred in Patient 24 during gene evolution. Patient 27 acquired a Notch1 mutation at the time of tumor recurrence. We speculate that the Notch1 mutation is not only a driver gene, but also may contribute to tumor progression and recurrence. TP53 mutation was persistent in both the primary tumor sample and the matched‐relapse TISF sample. In summary, our results indicated that oligodendroglioma has great heterogeneity during gene evolution and suggested that several well‐known signaling pathways and genes play an important role in tumor evolution, including the SHh signaling pathway, PI3K/AKT signaling pathway, Notch1 signaling pathway, and MMR proteins (MSH2 and MSH6).

### 3.3 Gene evolution in distant recurrence oligodendroglioma

**FIGURE 3:**
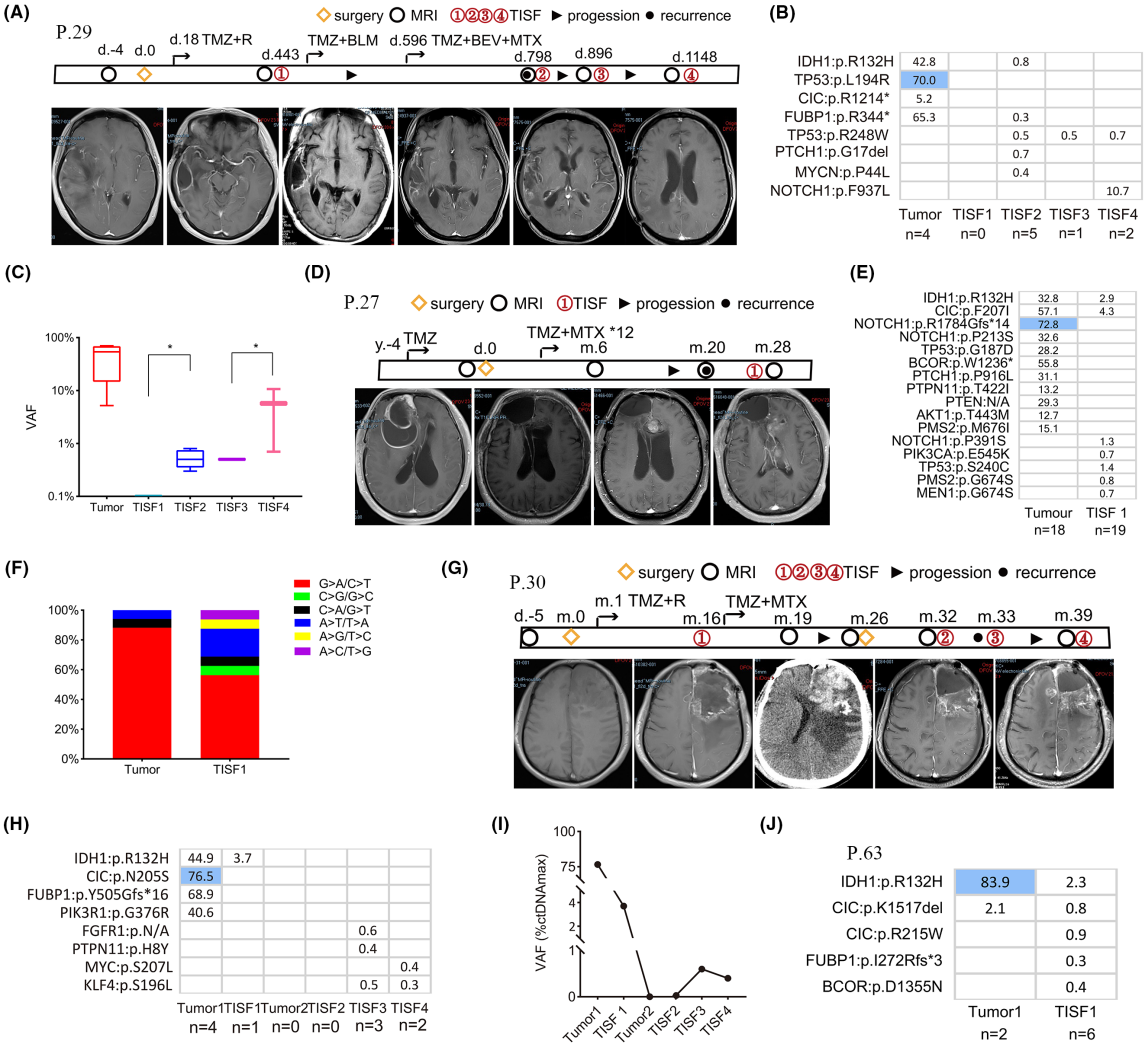
Gene evolution in distant relapse oligodendroglioma. (A) Clinical treatment, imaging changes, and the time of sample collection were shown (Patient 29). About 798 days later, imaging demonstrated local progression when TISF2 was detected. Distant relapse occurred at 1148 days after the operation, and TISF4 was collected. (B, C) Longitudinal clinical evolution and VAFs alternations were shown. Mean VAFs among the samples exhibit statistically significant differences. TISF2 sample had significantly higher VAF than that in TISF1 sample during the local progression (*p* < 0.01). Similarly, patient with distant recurrence also had higher VAF levels in TISF4 sample (*p* < 0.01). (D) Clinical and imaging alterations characteristics were depicted (Patient 27). Distant relapse occurred at 20 months after the operation. (E) Patient 27 displayed branched evolution with many clonal mutation alterations during tumor growth. (F) The proportion of TMZ‐associated mutations were identified in tumor and TISF samples. (G) Clinical, imaging, and therapeutic features of Patient 30 were depicted. Local relapse occurred 16 months after the operation. Ten months later, there was a second surgery due to sudden acute hematoma. Thirteen months later, he was diagnosed with a distant relapse. (H) Branched evolution of driver genes during progression was shown. (I) The maximum VAF statistically varied by tumor growth. (J) The gene evolution of Patient 63 was depicted during the progress of distant relapse.

Our study indicated a higher proportion of distant relapse during oligodendroglioma evolution. To elaborate on the evolution pattern of distant relapse, we investigated continuous tumor genomic evolution through a series of TISF‐ctDNA in vivo. Patient 29 was initially diagnosed with a lesion in the right temporal lobe by imaging, then underwent the surgery to resect the lesions, and was histopathologically diagnosed with anaplastic oligodendroglioma (WHO grade III). TP53:p.L194R, CIC:p.R1214*, and FUBP1:p.R344* mutations were detected as early driver genes, accompanied by IDH1:p.R132H mutant and 1p/19q‐co‐deleted. We collected TISF1 samples at 443 days after surgery, and none of the mutations with a VAF of ≥0.3% were detected. The result was consistent with the little progression of the tumor on imaging. The patient showed progressive symptoms 798 days after surgery, IDH1:p.R132H, TP53:p. R248W, PTCH1:p.G17del, and MYCN:p.P44L appeared in the TISF2 sample due to local progression. The patient continued to receive monthly TMZ, BEV, and MTX treatment. We collected TISF3 samples at 896 days after surgery and detected only one mutation (TP53:p.R248W) with a low VAF of 0.5%. The patient experienced distant recurrence in the corpus callosum approximately 3 years after surgery. NOTCH1:p.F937L with a VAF of 10.7% and TP53:p.R248W with a VAF of 0.7% were detected in the TISF4 sample (Figure 3A,B). As the disease progressed, the VAF value increased significantly (Figure 3C). It is obvious that oligodendroglioma displayed branched clonal evolution leading to tumor heterogeneity. Our study demonstrated that the emergence of subclones and IDH mutation status transformation promoted tumor malignancies and recurrence.

Patient 27 with a lesion in the right frontal lobe underwent surgery in 2014 and had a pathological diagnosis of oligodendroglioma (WHO grade II). The patient received long‐term TMZ treatment and underwent a second resection for a known local recurrence in 2018. The pathological diagnosis was anaplastic oligodendroglioma (WHO grade III). Gene analysis showed CIC:p.F207I, TP53: p.G187D, and IDH1:p.R132H as driver genes, while subclonal mutations in SHh (PTCH1:p.P916L), Wnt (PTEN:N/A), and PI3K/AKT signaling pathways were clearly detected in tissue, and MMR‐related subclone mutation was also found. PMS2 is a component of the post‐replicative DNA mismatch repair (MMR) system. MMR dysfunction ultimately led to TMZ resistance,^18^ and lead to a large number of C > T/G > A mutations (Figure 3F). Two years later, the distant recurrence progressed, PIK3CA:p.E545K was detected in the TISF sample. PIK3CA:p.E545K was one of the most common activation mutations and promoted tumor formation^19^ (Figure 3D,E). Oligodendroglioma displayed remarkable spatiotemporal heterogeneity during tumor evolution, and MMR dysfunction largely contributes to the accumulation of mutations during tumor progression.

Patient 30 had a lesion in the left frontal lobe and underwent the first surgery to resect the lesion in 2018. The pathological diagnosis was anaplastic oligodendroglioma (WHO grade III), with IDH1:p.R132H, PIK3R1:p.G376R, CIC:p.N205S, and FUBP1:p.Y505Gfs*16 mutations confirmed as driver genes in tumor tissue. We collected TISF1 samples 16 months after surgery and confirmed one mutation (IDH1:p.R132H) with a VAF of 3.7% associated with local tumor progression. The patient received TMZ combined with MTX treatment. Twenty‐six months after the first surgery, the patient underwent a second operation due to hematoma formation. However, our sequence analysis demonstrated that no gene mutations in the tissue were detected. We considered that the patient suffered from hemorrhage and did not appear to be due to tumor progression, and was likely to respond to chemotherapy. However, 39 months after the first surgery, the patient developed distant recurrence. Some subclonal mutations in PTCH1:p.R1350W and MYC:p.S207L appeared (Figure 3G,H). The MSAF of samples in the TISF samples increased as the tumor progressed (Figure 3I). Patient 63 acquired subclonal mutations in FUBP1:p.I271Rfs*3 and BCOR:p.D1355N when tumor recurrence was observed (Figure 3J). It can be seen that oligodendroglioma is sensitive to chemotherapy, but after long‐term TMZ treatment, oligodendroglioma displayed obvious spatiotemporal heterogeneity, that is, acquired a large number of subclonal mutations during gene evolution, which promotes tumor distant recurrence.

### 3.4 Distant relapse may be a later event in oligodendroglioma

**FIGURE 4:**
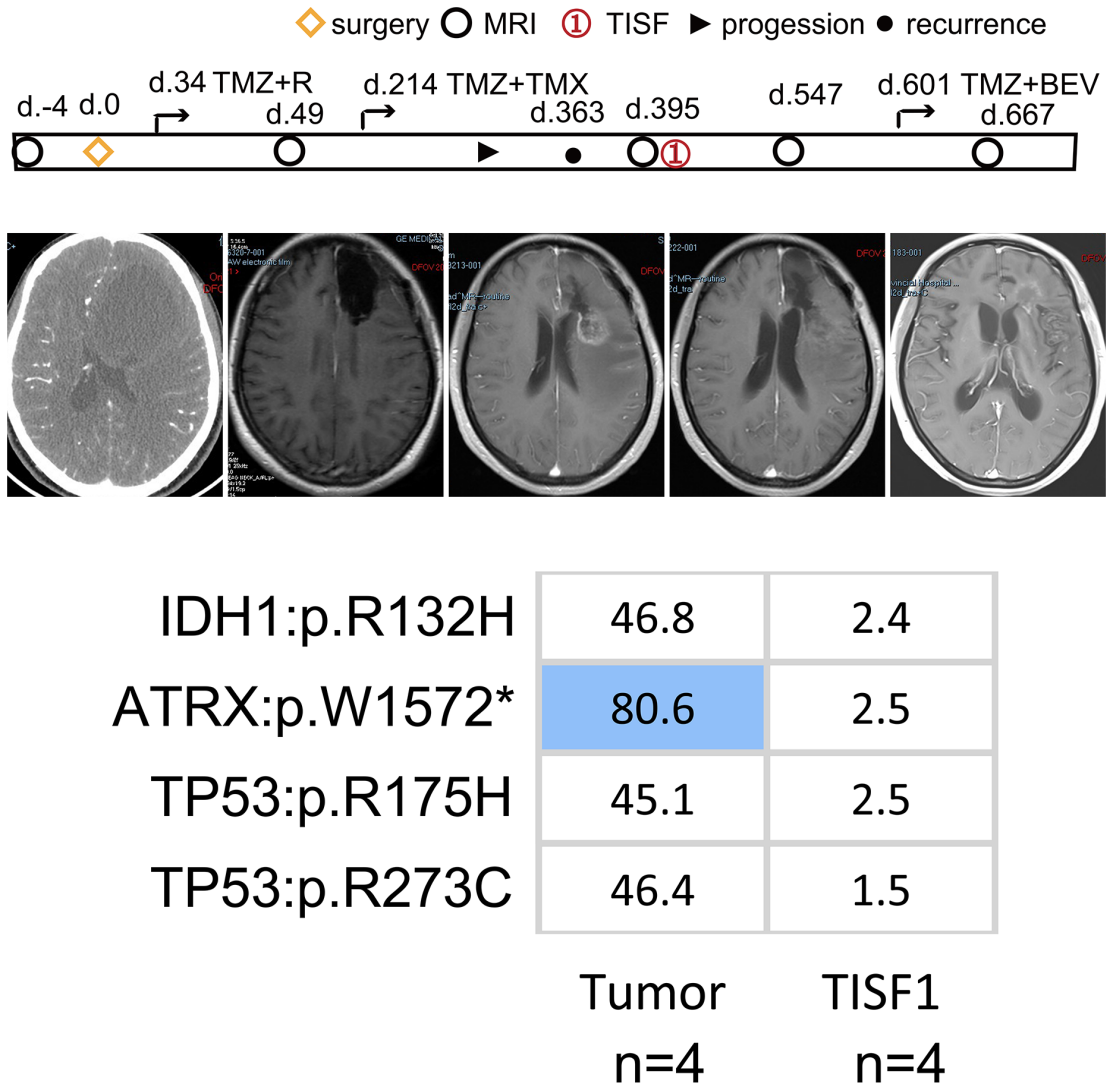
Molecular alterations during local progression in Patient 28. The patient had astrocytic or oligodendrocytic molecular features, received TMZ and radiotherapy treatment after gross total tumor resections. And the imaging analysis showed local progression 363 days after operation. Subsequently, TISF ctDNA was detected and harbored the same gene mutations that were detected in tissue. Under TMZ treatment, imaging demonstrated significant improvement.

Among the six recurrent oligodendrogliomas, one developed local recurrence. Patient 28 underwent surgical resection and was diagnosed with anaplasia oligodendroglioma (WHO grade III). IDH1:p.R132H mutation and 1p/19q co‐deletion were confirmed as driver events in the tumor tissue. Meanwhile, this patient had dominant mutations in TP53:p.R175H, TP53:p.R273C, and ATRX:p.W1572*. One year after the operation, imaging demonstrated local progression, and TISF1 sample was harvested at the time. We observed that the same mutations were present in both the initial and recurrent samples (Figure 4). The TP53:p.R175H mutation is a functional acquired mutation closely related to the high invasiveness and metastatic potential of many cancers.^20^ TP53:p.R273C is also a functional acquired mutation that promotes the proliferation, invasion, and drug resistance of tumor cells.^21^ The 1p/19q co‐deletion is positively associated with IDH mutations, while it is mutually exclusive with ATRX loss and TP53 mutation, which are the hallmarks of diffuse astrocytoma.^22^ Patient 28 had the molecular characteristics of oligodendroglioma and the molecular information of astrocytoma, and there was no tendency for an increase in mutation numbers. Subsequently, the local lesion improved significantly under TMZ treatment. Similarly, Patients 29 and 30 did not experience changes in IDH mutation status when local progression occurred (Figure 3B,H). However, under long‐term TMZ chemotherapy, IDHmt‐to‐IDHwt transformation and additional subclonal mutations were present when distant relapse occurred and had a tendency to rapid tumor progression, indicating that the cell phenotype and genetic characteristics of oligodendroglioma will change under TMZ treatment. Oligodendroglioma not only has the characteristics of oligodendrocyte, but also has the molecular characteristics of astrocytoma in the samples from patient 28. The IDH mutation transformation suggests that relapse may emerge in the later stages of tumor evolution after diverging from the initial tumor ancestors. Distant relapse may be a later event during oligodendroglioma evolution.

### 3.5 Hypermutation in oligodendroglioma

**FIGURE 5:**
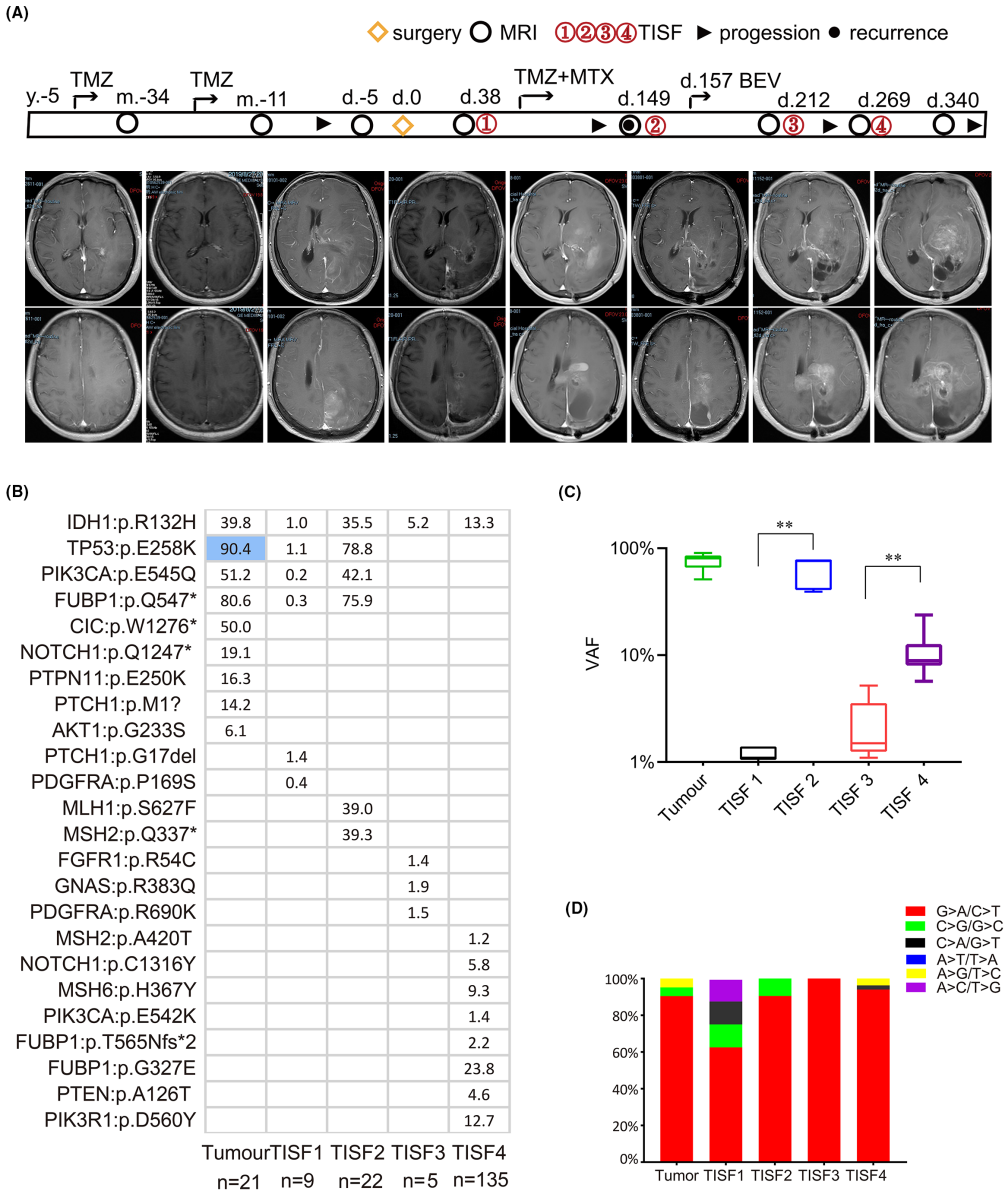
Hypermutation in oligodendroglioma. (A) ctDNA extraction, imaging and therapeutic features of Patient 24 were showed during the tumor progression. About 269 days after the operation, hypermutation occurred in TISF4 sample. (B) Distribution of mutations in tumor and TISF samples. The acquired mutations and number alterations were depicted. (C) Mean VAFs among the top 5% exhibited statistically significant differences in tumor and TISF samples. TISF2 sample had higher VAF than that in TISF1 sample during tumor progression (*p* < 0.01), and TISF4 sample had significantly higher VAF than that in TISF3 sample (*p* < 0.01). (D) The proportion of TMZ‐associated mutations was calculated in tumor and TISF samples. This proportion was 90.5% (tumor), 62.5% (TISF1), 90.5% (TISF2), 100% (TISF3), and 94.0% (TISF4), respectively.

Hypermutated tumors have been reported in patients with glioblastomas and astrocytomas,^23^ but hypermutation has not previously been reported in the oligodendroglioma patients, even after 12 courses of TMZ chemotherapy.^9^ However, Patient 24 who had MMR‐associated mutations, exhibited a hypermutable phenotype, which may have been related to TMZ treatment. Patient 24 was initially diagnosed with a lesion in 2015 and received long‐term TMZ treatment. Unfortunately, multiple lesions were detected by imaging in 2020, and the patient underwent surgery, during which the tumor was diagnosed as oligodendroglioma (WHO grade III). We confirmed IDH, Notch1, CIC, and FUBP1 as the driver genes, accompanied by 1p/19q co‐deletion (Figure 3E). Multiple events in signaling pathways could be seen (e.g., SHh, PI3K/AKT, and YAP1/Hippo signaling pathways), which contributed to the biological characteristics of distant relapse. The tumor remained stable, and accordingly, all mutations were detected with a VAF less than 1.5% in TISF1 samples. However, imaging revealed an increase in tumor burden at 138 days post‐operation. Additionally, an MMR mutation in MSH2:p.Q337* was detected in the TISF2 sample, and the average VAFs in the TISF2 sample were significantly higher than those in the TISF1 sample. The patient then received TMZ and BEV treatment starting on day 157. The tumor load was significantly reduced, and the mutational burden decreased in the TISF3 sample. However, progression was noted again at 269 days after surgery, at which time, hypermutation occurred and 135 mutations were detected in the TISF4 sample (Figure 5B). And MSH6:p.H367Y and MSH2:p.A420T were included in the gene panel, and hypermutated tumor was more likely to progress rapidly and widely. The mean VAF in TISF4 was significantly increased again (Figure 5C). In addition, the proportion of total TMZ‐associated mutations in the TISF4 sample was 94% (Figure 5D). There is evidence to suggest that the hypermutation is a contributor to malignant progression that can occur during the process of human oligodendroglioma evolution.

## 4 DISCUSSION

In our study, we investigated the gene evolution of oligodendroglioma under the TMZ treatment. The retention rate during tumor evolution was found to be low, with a mean retention rate of 26.8%. The trunk genes showed changes in two patients, indicating that oligodendroglioma exhibited a complicated branching evolutionary pattern. We demonstrated that subclonal expansion led to the progression and deterioration of the tumor, with five out of six patients developing distant recurrence. Furthermore, we found that distant relapse occurred later than local progression, suggesting that distant recurrence may be a later event during tumor evolution. FUBP1 is a transcription regulator, FUBP1 mutation leads to MYC activation by relieving the negative effects of the FUBP1‐PUF60‐FUSE complex.^5^ FUBP1 mutation may provide a survival advantage to tumor cells. However, we found that potential driver gene mutations, such as CIC and FUBP1 mutations, were not maintained in the relapse samples of Patients 29 and 30 studied. This suggests that mutations in CIC and FUBP1 may have a weaker effect on tumor progression. Mutations in the promoter region of TERT are common driver events in oligodendrogliomas, but they were not detectable due to the limitations of exome sequencing. Therefore, we were unable to assess the effect of TERT promoter alterations on evolution processes. But the Notch1 gene has been widely assumed to be a driver gene in oligodendroglioma. In our study, Notch1: p.Q1247* and Notch1:p.R1784Gfs*14 were detected in Patients 24 and 27, respectively, under long‐term TMZ treatment. Notch1 pathway globally reduced oligodendrocytic markers and IDH1 expression while upregulating APOE, CRYAB, and HEY1/2, was accompanied by a reduction in cell proliferation. Notch1 signaling pathway is known to act as a tumor suppressor in IDH‐DGIIG.^24^ Patient 29 acquired a Notch1:p.F937L mutation that was accompanied by a transformation in IDH mutation status during tumor evolution. Notch1 signaling was activated in TGFβ2‐stimulated EMT, and played a significant role in cancer metastasis and recurrence.^25^ Therefore, we speculate that the Notch1 signaling pathway may be responsible for cancer resistance and recurrence during malignant progression.

After long‐term TMZ chemotherapy, Patient 24 had multiple lesions, and mutations in the PI3K/AKT signaling pathway were found, such as AKT1:p.G233S, PIK3CA:p.E545Q, and PTPN11:p.E250K. Similarly, Patient 27 had mutations closely related to the PI3K/AKT signaling pathway following long‐time TMZ chemotherapy, such as AKT1:p.T443M and PTPN11:p.T422I. Two years after the operation, multiple lesions were observed in the image, and PIK3CA:p.E545K mutation was detected in TISF sample. Previous studies have reported that PI3K/AKT signaling pathway drived malignant progression of oligodendrogliomas.^26^ PIK3CA activating mutations have been associated with earlier recurrence and shorter survival in adult glioblastoma.^27^ The PI3K/AKT signaling pathway has been shown to promote cancer progression and distant recurrence,^28^, ^29^ enhance resistance to TMZ, and and govern cancer stem cell stemness.^30^, ^31^, ^32^ Therefore, PI3K/AKT signaling alteration promotes malignant progression and distant relapse of oligodendrogliomas.

Patient 29 acquired the PTCH1:p.G17del mutation during the process of tumor progression. Patient 30 acquired the PTCH1:R1350W mutation when distant relapse occurred. Patients 24 and 27 also obtained PTCH1 mutations after undergoing long‐term TMZ chemotherapy. This further suggested that PTCH1 mutations may be responsible for the progression of oligodendroglioma into its more aggressive form. The PTCH1 gene, a tumor suppressor gene, encodes an inhibitory transmembrane protein receptor that continuously inhibits the activation of the Hedgehog (SHh) signaling pathway by inhibiting SMO receptor activity.^33^ The SHh signaling pathway, which is continuously activated due to inactivation mutations in the PTCH1 gene, is commonly observed in basal cell carcinoma and medulloblastoma.^34^, ^35^ Furthermore, PTCH1 mutations have also been linked to the promotion of distant metastasis and recurrence in basal cell carcinoma.^35^ Hedgehog (SHh) signal endows tumors with high invasiveness,^36^ and its aberrant activation drives tumorigenesis in various cancers, including glioblastoma.^37^ Recently, the SHh pathway was found to possess a key function in the progression and metastasis of various cancers.^38^, ^39^, ^40^ We believed that the SHh signaling pathway has been shown to be vital role in promoting malignant progression in vivo and distant relapse during oligodendroglioma evolution.

IDH‐mutant oligodendrogliomas are slow‐growing brain tumors that may progress into high‐grade gliomas. The IDH mutation status remained unchanged in Patients 29 and 30 with locally advanced tumors. However, further TMZ treatments, gene analysis demonstrated the transition of tumor cells from IDH mutation states into wild states when distant relapse occurred. These results imply that the cell phenotype may change during tumor evolution. Based on gene evolution in vivo, it is clear that low‐grade glioma can progress into a more aggressive tumor. Oligodendroglioma originates from oligodendrocytes and glial precursor cells.^41^ The IDH mutation transformation suggests that recurrences may emerge in the later stages of tumor evolution after diverging from the initial tumor ancestors.

A high percentage of O6‐methylguanine‐DNA methyltransferase (MGMT) methylation and low expression of DNA repair genes mediate sensitivity to temozolomide.^42^ TMZ‐induced hypermutation is a common event in transformed LGG previously treated with TMZ treatment.^43^ The hypermutation phenotype is thought to be related to the response to TMZ treatment. Defects in the mismatch repair system lead to a large number of C > T/G > A mutations, which have a tendency to induce hypermutation and resist TMZ therapy.^44^ Oligodendroglioma can also acquire a hypermutator phenotype as a result of TMZ‐based chemotherapy. Patient 24 received long‐term TMZ chemotherapy before surgery, and TMZ chemotherapy was continued postoperatively. Mutations in MSH2 and MSH6 were detected and hypermutation was observed 9 months after the surgery, which was associated with rapid tumor progression. Understanding the dynamics of tumor evolution can help in the development of future therapeutic strategies against cancer. In our study, liquid biopsies may monitor the dynamic evolution of the glioma genome as a minimally invasive alternative. We have reported for the first time the real‐time evolution of oligodendroglioma in vivo under the TMZ adjuvant chemotherapy. Given the limitations of exome sequencing, we acknowledge the obvious disadvantages of this analysis. It is likely that we were unable to elaborate on evolution information for some important driver events, such as TERT promoter mutation, 1p/19q co‐deletion, and MGMT promoter methylation. Further research strategies should focus on the disadvantages described above.

## 5 CONCLUSIONS

In conclusion, our results demonstrated that oligodendroglioma has great heterogeneity during gene evolution, which leads to the rapid progression of the tumor. Distant relapse may occur as a later event during oligodendroglioma progression. Our findings indicate that the PI3K/AKT and SHh signaling pathways may play important roles in promoting tumor distant relapse and drug resistance during oligodendroglioma progression. The IDH mutation transformation and hypermutation observed in our study suggest that the cell phenotype and genetic characteristics of oligodendroglioma can also change during tumor evolution, leading to an increased malignant degree.

## AUTHOR CONTRIBUTIONS

**Guanzheng Liu:** Conceptualization (equal); formal analysis (equal); funding acquisition (equal); methodology (equal); writing – original draft (lead). **Chaojie Bu:** Conceptualization (equal); data curation (equal); investigation (equal); methodology (equal). **Guangzhong Guo:** Investigation (equal); methodology (equal); resources (equal); software (equal). **Zhiyue Zhang:** Investigation (equal); methodology (equal); resources (equal); software (equal). **Zhiyuan Sheng:** Conceptualization (equal); investigation (equal); methodology (equal); software (equal). **Kaiyuan Deng:** Data curation (equal); investigation (equal); methodology (equal); resources (equal); software (equal). **Shuang Wu:** Investigation (equal); methodology (equal); resources (equal); software (equal). **Sensen Xu:** Data curation (equal); investigation (equal); methodology (equal). **Yage Bu:** Investigation (equal); methodology (equal); resources (equal); software (equal). **Yushuai Gao:** Investigation (equal); methodology (equal). **Meiyun Wang:** Resources (equal); validation (equal); visualization (equal). **Gang Liu:** Resources (equal); software (equal); validation (equal); visualization (equal). **Lingfei Kong:** Software (equal); supervision (equal); visualization (equal). **Tianxiao Li:** Supervision (equal); validation (equal); visualization (equal). **Ming Li:** Resources (equal); software (equal); supervision (equal); validation (equal). **Xingyao Bu:** Funding acquisition (equal); project administration (equal); supervision (equal); visualization (equal); writing – review and editing (equal).

## FUNDING INFORMATION

The research was supported by the Henan Province Science and Technology Tackle Program (No.192102310126), the Joint Project of Medical Science and Technology Tackling Plan of Henan Province (No.201601016), and the Henan Medical Science and Technology Research Youth Co‐construction Project (No. SJGJ202103017).

## CONFLICT OF INTEREST STATEMENT

The authors declare that they have no competing interests.

## ETHICS STATEMENT

All experimental protocols were approved by the Ethics Committee of the Zhengzhou University (approval number 2122432055).

## CONSENT STATEMENT

Not applicable.

## Supporting information


Figure S1.

Table S1.

## Data Availability

Datasets supporting the conclusions contained in the present report are included in the manuscript all data and supplementary material. The sequencing data will be publicly available in the NCBI repository (PRJNA962034). The sequencing information can be found online at https://dataview.ncbi.nlm.nih.gov/object/PRJNA962034.

## References

[1] Louis DN , Perry A , Wesseling P , et al. The 2021 WHO classification of tumors of the central nervous system: a summary. Neuro Oncol. 2021;23(8):1231‐1251. 34185076 10.1093/neuonc/noab106 PMC8328013

[2] Lavon I , Zrihan D , Zelikovitch B , et al. Longitudinal assessment of genetic and epigenetic markers in oligodendrogliomas. Clin Cancer Res. 2007;13(5):1429‐1437. 17332285 10.1158/1078-0432.CCR-06-2050

[3] Cairncross JG , Macdonald DR . Successful chemotherapy for recurrent malignant oligodendroglioma. Ann Neurol. 1988;23(4):360‐364. 3382171 10.1002/ana.410230408

[4] Chinot O . Chemotherapy for the treatment of oligodendroglial tumors. Semin Oncol. 2001;28(4 Suppl 13):13‐18. 10.1016/s0093-7754(01)90066-1 11550134

[5] Bettegowda C , Agrawal N , Jiao Y , et al. Mutations in CIC and FUBP1 contribute to human oligodendroglioma. Science. 2011;333(6048):1453‐1455. 21817013 10.1126/science.1210557 PMC3170506

[6] Kanamori M , Kumabe T , Shibahara I , et al. Clinical and histological characteristics of recurrent oligodendroglial tumors: comparison between primary and recurrent tumors in 18 cases. Brain Tumor Pathol. 2013;30(3):151‐159. 23053495 10.1007/s10014-012-0119-8

[7] Burrell RA , McGranahan N , Bartek J , Swanton C . The causes and consequences of genetic heterogeneity in cancer evolution. Nature. 2013;501(7467):338‐345. 24048066 10.1038/nature12625

[8] Mazor T , Pankov A , Johnson BE , et al. DNA methylation and somatic mutations converge on the cell cycle and define similar evolutionary histories in brain tumors. Cancer Cell. 2015;28(3):307‐317. 26373278 10.1016/j.ccell.2015.07.012 PMC4573399

[9] Aihara K , Mukasa A , Nagae G , et al. Genetic and epigenetic stability of oligodendrogliomas at recurrence. Acta Neuropathol Commun. 2017;5(1):18. 28270234 10.1186/s40478-017-0422-z PMC5339990

[10] Wu L , Ou Y , Liu B , Liu W . Scalp metastasis of anaplastic oligodendroglioma. World Neurosurg. 2019;128:448‐451. 31125773 10.1016/j.wneu.2019.05.109

[11] Burgy M , Chenard MP , Noel G , Bourahla K , Schott R . Bone metastases from a 1p/19q codeleted and IDH1‐mutant anaplastic oligodendroglioma: a case report. J Med Case Reports. 2019;13(1):202. 10.1186/s13256-019-2061-4 PMC6598291 31248444

[12] Sheng Z , Yu J , Deng K , et al. Characterizing the genomic landscape of brain glioma with circulating tumor DNA from tumor In situ fluid. Front Oncol. 2021;11:584988. 33868989 10.3389/fonc.2021.584988 PMC8045748

[13] Yu J , Sheng Z , Wu S , et al. Tumor DNA from tumor In situ fluid reveals mutation landscape of minimal residual disease after glioma surgery and risk of early recurrence. Front Oncol. 2021;11:742037. 34712610 10.3389/fonc.2021.742037 PMC8547270

[14] Chan AK , Pang JC , Chung NY , et al. Loss of CIC and FUBP1 expressions are potential markers of shorter time to recurrence in oligodendroglial tumors. Mod Pathol. 2014;27(3):332‐342. 24030748 10.1038/modpathol.2013.165

[15] Avliyakulov NK , Rajavel KS , Le KM , et al. C‐terminally truncated form of alphaB‐crystallin is associated with IDH1 R132H mutation in anaplastic astrocytoma. J Neurooncol. 2014;117(1):53‐65. 24473683 10.1007/s11060-014-1371-z

[16] Gleize V , Alentorn A , Connen de Kerillis L , et al. CIC inactivating mutations identify aggressive subset of 1p19q codeleted gliomas. Ann Neurol. 2015;78(3):355‐374. 26017892 10.1002/ana.24443

[17] Kim J , Lee IH , Cho HJ , et al. Spatiotemporal evolution of the primary glioblastoma genome. Cancer Cell. 2015;28(3):318‐328. 26373279 10.1016/j.ccell.2015.07.013

[18] Aasland D , Gotzinger L , Hauck L , et al. Temozolomide induces senescence and repression of DNA repair pathways in glioblastoma cells via activation of ATR‐CHK1, p21, and NF‐kappaB. Cancer Res. 2019;79(1):99‐113. 30361254 10.1158/0008-5472.CAN-18-1733

[19] Pirozzi F , Berkseth M , Shear R , et al. Profiling PI3K‐AKT‐MTOR variants in focal brain malformations reveals new insights for diagnostic care. Brain. 2022;145(3):925‐938. 35355055 10.1093/brain/awab376 PMC9630661

[20] Ikeda H , Kukitsu T , Johmen W , et al. Gastric invasive micropapillary carcinoma with intestinal phenotypes harboring a TP53 R175H mutation. Case Rep Oncol. 2014;7(3):611‐620. 25408652 10.1159/000367583 PMC4209268

[21] Muller PA , Vousden KH . Mutant p53 in cancer: new functions and therapeutic opportunities. Cancer Cell. 2014;25(3):304‐317. 24651012 10.1016/j.ccr.2014.01.021 PMC3970583

[22] Wiestler B , Capper D , Holland‐Letz T , et al. ATRX loss refines the classification of anaplastic gliomas and identifies a subgroup of IDH mutant astrocytic tumors with better prognosis. Acta Neuropathol. 2013;126(3):443‐451. 23904111 10.1007/s00401-013-1156-z

[23] Johnson BE , Mazor T , Hong C , et al. Mutational analysis reveals the origin and therapy‐driven evolution of recurrent glioma. Science. 2014;343(6167):189‐193. 24336570 10.1126/science.1239947 PMC3998672

[24] Augustus M , Pineau D , Aimond F , et al. Identification of CRYAB(+) KCNN3(+) SOX9(+) astrocyte‐like and EGFR(+) PDGFRA(+) OLIG1(+) oligodendrocyte‐like tumoral cells in diffuse IDH1‐mutant gliomas and implication of NOTCH1 Signalling in their genesis. Cancers (Basel). 2021;13(9):2107. 33925547 10.3390/cancers13092107 PMC8123787

[25] Chen X , Xiao W , Chen W , et al. MicroRNA‐26a and ‐26b inhibit lens fibrosis and cataract by negatively regulating Jagged‐1/notch signaling pathway. Cell Death Differ. 2017;24(8):1431‐1442. 28622289 10.1038/cdd.2016.152 PMC5520447

[26] Tateishi K , Nakamura T , Juratli TA , et al. PI3K/AKT/mTOR pathway alterations promote malignant progression and xenograft formation in Oligodendroglial tumors. Clin Cancer Res. 2019;25(14):4375‐4387. 30975663 10.1158/1078-0432.CCR-18-4144 PMC6924174

[27] Tanaka S , Batchelor TT , Iafrate AJ , et al. PIK3CA activating mutations are associated with more disseminated disease at presentation and earlier recurrence in glioblastoma. Acta Neuropathol Commun. 2019;7(1):66. 31036078 10.1186/s40478-019-0720-8 PMC6487518

[28] Ma XL , Shen MN , Hu B , et al. CD73 promotes hepatocellular carcinoma progression and metastasis via activating PI3K/AKT signaling by inducing Rap1‐mediated membrane localization of P110beta and predicts poor prognosis. J Hematol Oncol. 2019;12(1):37. 30971294 10.1186/s13045-019-0724-7 PMC6458749

[29] Wei C , Dong X , Lu H , et al. LPCAT1 promotes brain metastasis of lung adenocarcinoma by up‐regulating PI3K/AKT/MYC pathway. J Exp Clin Cancer Res. 2019;38(1):95. 30791942 10.1186/s13046-019-1092-4 PMC6385475

[30] Zhang LH , Yin AA , Cheng JX , et al. TRIM24 promotes glioma progression and enhances chemoresistance through activation of the PI3K/Akt signaling pathway. Oncogene. 2015;34(5):600‐610. 24469053 10.1038/onc.2013.593

[31] Qin Y , Hou Y , Liu S , et al. A novel long non‐coding RNA lnc030 maintains breast cancer stem cell stemness by stabilizing SQLE mRNA and increasing cholesterol synthesis. Adv Sci (Weinh). 2021;8(2):2002232. 33511005 10.1002/advs.202002232 PMC7816696

[32] Yang L , Shi P , Zhao G , et al. Targeting cancer stem cell pathways for cancer therapy. Signal Transduct Target Ther. 2020;5(1):8. 32296030 10.1038/s41392-020-0110-5 PMC7005297

[33] Huang P , Wierbowski BM , Lian T , et al. Structural basis for catalyzed assembly of the sonic hedgehog‐Patched1 signaling complex. Dev Cell. 2022;57(5):670‐685 e678. 35231446 10.1016/j.devcel.2022.02.008 PMC8932645

[34] Coltin H , Sundaresan L , Smith KS , et al. Subgroup and subtype‐specific outcomes in adult medulloblastoma. Acta Neuropathol. 2021;142(5):859‐871. 34409497 10.1007/s00401-021-02358-4 PMC10723183

[35] Verkouteren BJA , Wakkee M , van Geel M , et al. Molecular testing in metastatic basal cell carcinoma. J Am Acad Dermatol. 2021;85(5):1135‐1142. 31870915 10.1016/j.jaad.2019.12.026

[36] Fan YH , Ding J , Nguyen S , et al. Aberrant hedgehog signaling is responsible for the highly invasive behavior of a subpopulation of hepatoma cells. Oncogene. 2016;35(1):116‐124. 25772244 10.1038/onc.2015.67

[37] Wu X , Xiao S , Zhang M , et al. A novel protein encoded by circular SMO RNA is essential for hedgehog signaling activation and glioblastoma tumorigenicity. Genome Biol. 2021;22(1):33. 33446260 10.1186/s13059-020-02250-6 PMC7807754

[38] Lee DH , Lee SY , Oh SC . Hedgehog signaling pathway as a potential target in the treatment of advanced gastric cancer. Tumour Biol. 2017;39(6):1010428317692266. 28621241 10.1177/1010428317692266

[39] Szczepny A , Rogers S , Jayasekara WSN , et al. The role of canonical and non‐canonical hedgehog signaling in tumor progression in a mouse model of small cell lung cancer. Oncogene. 2017;36(39):5544‐5550. 28581526 10.1038/onc.2017.173 PMC5623150

[40] Ishaque N , Abba ML , Hauser C , et al. Whole genome sequencing puts forward hypotheses on metastasis evolution and therapy in colorectal cancer. Nat Commun. 2018;9(1):4782. 30429477 10.1038/s41467-018-07041-z PMC6235880

[41] Gimenez M , Marie SK , Oba‐Shinjo S , et al. Quantitative proteomic analysis shows differentially expressed HSPB1 in glioblastoma as a discriminating short from long survival factor and NOVA1 as a differentiation factor between low‐grade astrocytoma and oligodendroglioma. BMC Cancer. 2015;15:481. 26108672 10.1186/s12885-015-1473-9 PMC4502388

[42] Ruda R , Touat M , Soffietti R . Is chemotherapy alone an option as initial treatment for low‐grade oligodendrogliomas? Curr Opin Neurol. 2020;33(6):707‐715. 33027142 10.1097/WCO.0000000000000866

[43] Yu Y , Villanueva‐Meyer J , Grimmer MR , et al. Temozolomide‐induced hypermutation is associated with distant recurrence and reduced survival after high‐grade transformation of low‐grade IDH‐mutant gliomas. Neuro Oncol. 2021;23(11):1872‐1884. 33823014 10.1093/neuonc/noab081 PMC8563321

[44] Mathur R , Zhang Y , Grimmer MR , et al. MGMT promoter methylation level in newly diagnosed low‐grade glioma is a predictor of hypermutation at recurrence. Neuro Oncol. 2020;22(11):1580‐1590. 32166314 10.1093/neuonc/noaa059 PMC8444710

