# Supplementary material for: Genomic alterations of oligodendrogliomas at distant recurrence
Source: Cancer Med. 2023 Aug 2;12(16):17171–83. doi: 10.1002/cam4.6327 (PMC10501240; doi:10.1002/cam4.6327)
Supplement: Supplementary file 1 — Figure S1. [file CAM4-12-17171-s001.docx]

Fig. S1 Mutational landscape of glioma in the tumors and TISF in 11

oligodendrogliomas.
