# Supplementary material for: Genomic alterations of oligodendrogliomas at distant recurrence
Source: Cancer Med. 2023 Aug 2;12(16):17171–83. doi: 10.1002/cam4.6327 (PMC10501240; doi:10.1002/cam4.6327)
Supplement: Supplementary file 2 — Table S1. [file CAM4-12-17171-s002.docx]

Table S1: Clinical characteristics of the respective patient.

| Patient no | Sex | Age | Histopathology | WHO grade | Location | Relapsed | Treatment |
| --- | --- | --- | --- | --- | --- | --- | --- |
| 22 | M | 46 | O | II | frontal | NO | CT |
| 23 | M | 47 | O | II | temporal | NO | CT |
| 24 | F | 57 | AO | III | parietal | MR | CT |
| 27 | M | 47 | AO | III | frontal | MR | CT |
| 28 | F | 46 | AO | III | frontal | LR | CT+RT |
| 29 | F | 52 | AO | III | temporal | MR | CT+RT |
| 30 | M | 56 | AO | III | frontal | MR | CT+RT |
| 43 | F | 61 | AO | III | temporal | NO | CT+RT |
| 63 | F | 42 | O | II | frontal | MR | CT |
| 64 | M | 34 | O | II | frontal | NO | CT |
| 69 | F | 35 | AO | III | frontal | NO | CT+RT |

F:female; M:male; O:oligodendroglioma; AO, anaplastic oligodendroglioma; CT: chemotherapy; RT: Radiotherapy ;LR: local relapsed; MR: metastasis recurrence;-:none or unknown
